# How can GPS technology help us better understand exposure to the food environment? A systematic review

**DOI:** 10.1016/j.ssmph.2016.04.001

**Published:** 2016-04-18

**Authors:** Andreea Cetateanu, Andy Jones

**Affiliations:** a School of Environmental Sciences, University of East Anglia, Norwich, Norfolk NR4 7TJ, UK; b Norwich Medical School, University of East Anglia, Norwich, Norfolk NR4 7TJ, UK; c Centre for Diet and Physical Activity Research, Box 296, Institute of Public Health, Forvie Site, Robinson Way, Cambridge CB2 0SR, UK; d School of Public Health, Imperial College London, St Mary׳s Campus, Medical School Building, Office G39, Norfolk Place, W2 1PG London, UK

**Keywords:** Global positioning systems, Geographic information system, Food environments, Food exposure, Systematic review

## Abstract

**Purpose:**

Global Positioning Systems (GPS) are increasingly being used to objectively assess movement patterns of people related to health behaviours. However research detailing their application to the food environment is scarce. This systematic review examines the application of GPS in studies of exposure to food environments and their potential influences on health.

**Methods:**

Based on an initial scoping exercise, published articles to be included in the systematic review were identified from four electronic databases and reference lists and were appraised and analysed, the final cut-off date for inclusion being January 2015. Included studies used GPS to identify location of individuals in relation to food outlets and link that to health or diet outcomes. They were appraised against a set of quality criteria.

**Results:**

Six studies met the inclusion criteria, which were appraised to be of moderate quality. Newer studies had a higher quality score. Associations between observed mobility patterns in the food environment and diet related outcomes were equivocal. Findings agreed that traditional food exposure measures overestimate the importance of the home food environment.

**Conclusions:**

The use of GPS to measure exposure to the food environment is still in its infancy yet holds much potential. There are considerable variations and challenges in developing and standardising the methods used to assess exposure.

## Highlights

• Since 2011 just 6 studies have been published employing GPS to quantify exposure to food environments.
• The studies were generally only of moderate quality.
• There were significant variations and challenges in the methods and techniques used.
• GIS neighbourhoods may not well represent food environment exposures based on GPS data.

## 1 Introduction

### 1.1 Understanding the food environment, its use and the link with health related outcomes and behaviours

Environmental factors have been shown to influence health behaviours (Ball, Timperio, & Crawford, 2006), and understanding their importance has formed a growing area of research, driven by the emergence of social-ecological theory and a shift of focus from individual-level influences on health (Stokols, 1992, Stokols, 2000). One area of particular interest has been the influence of the macro-level food environment on weight and associated dietary behaviours, food intake, and food purchasing (Bader et al., 2010, Burgoine et al., 2013).

Motivated by concerns over rising obesity prevalence (Cetateanu and Jones, 2014, de Onis et al., 2010, National_Obesity_Observatory National Child Measurement Programme, 2013), researchers have begun mapping exposure to the food environment and relating it to relevant health outcomes. The food environment, broadly conceptualised to include any opportunity to obtain food, can encompass a variety of features, such as availability and accessibility to outlets selling food (Lake & Townshend, 2006) in the residential, school, work, or activity spaces, with the latter defining the places people go to purchase food or the food they are exposed to while doing their daily activities (Christian, 2012). There are various hypotheses that link these food environments to diet, weight, and other health-related outcomes (An & Sturm, 2012), either directly or through the influence of other factors such as socio-economic status (Cetateanu & Jones, 2014). Yet, despite the fact that conceptually it is evident that less supportive environments for health eventually lead to worse diets and elevated weight, the findings reported in the literature are equivocal (An and Sturm, 2012, Boone-Heinonen et al., 2011, Pearce et al., 2008, Sturm and Datar, 2005, Wang et al., 2007), with studies reporting mixed associations between various food environment exposure measures and health outcomes (Christian, 2012, Gustafson et al., 2013, Zenk et al., 2011).

Some studies find associations with some relevant outcomes such as overweight and/or obesity (Cetateanu and Jones, 2014, Fraser and Edwards, 2010) or certain types of food consumption (e.g. fast food) (Burgoine, Forouhi, Griffin, Wareham, & Monsivais, 2014), whilst others find none with consumption of different food types (An & Sturm, 2012) or with BMI (An & Sturm, 2012) or overweight (Burdette & Whitaker, 2004) or obesity (Simmons et al., 2005). It is pertinent that two systematic reviews on the environment and obesity suggest that the great heterogeneity across studies limits what can be learned from this body of evidence (Feng et al., 2010, Holsten, 2009). It has recently been suggested that such equivocal results might be because of imprecision in measurement of exposure to the environment; for example, facilities being present in an area does not necessarily mean that people will use them. Further, it is often challenging to draw a categorical distinction between what is a ׳healthy’ and what is an ׳unhealthy’ food outlet, as the majority of food outlets sell items which vary in their healthfulness. It has therefore been suggested that a distinction should be made between the ‘community food environment’ vs. the ‘consumer food environment’ (Glanz, Sallis, Saelens, & Frank, 2005), which entails distinguishing the measurement of stores from the measurement of foods purchased and consumed (Caspi, Sorensen, Subramanian, & Kawachi, 2012).

Researchers are increasingly using geospatial technologies (Kerr et al., 2011, Hillier, 2008) to model the environment or how people interact with it. These include GIS (geographical information systems) (Moore, Diez Roux, Nettleton, Jacobs, & Franco, 2009), global positioning systems (GPS) (Zenk et al., 2011), smartphones (Boulos and Yang, 2013, Iverson,), tablets (Boulos & Yang, 2013), PDAs (handheld personal digital assistants) (Fitzgerald, 2005), Google Maps (Wang et al., 2011) and smart card technology (Lambert et al., 2005). Much of the evidence in the literature is however based on the use of GIS to compute measures of assumed exposures to the food environment based on the location of facilities (Burgoine et al., 2013) and typically focused on residential neighbourhoods with indicators of proximity/density used to describe retail food accessibility (Christian, 2012). Despite their popularity, these methods have several limitations. In particular, they typically fail to account for daily movements of individuals. This is pertinent given that it has been shown that people conduct only a small proportion of their daily activity within the residential neighbourhood (Hillsdon et al., 2015, Inagami et al., 2006). As a result, arguments have been made of the need for future research to consider food environments outside of residential neighbourhoods and also to consider how individuals interact with these environments (Papas et al., 2007). This has led to a recent increase in studies using GPS (Boruff, Nathan, Nijënstein, & Using, 2012) applied to looking at the ‘activity space’ of people by tracking their mobility patterns (Kerr et al., 2011, Thornton et al., 2011).

### 1.2 What does GPS contribute?

GPS is a satellite-based global navigation system that provides an accurate location of any point on the Earth׳s surface (Krenn, Titze, Oja, Jones, & Ogilvie, 2011). It thus provides a means to objectively assess the spatial location of features in the environment or people׳s behaviours while moving in the environment. Outdoor GPS rely on being able to receive a signal from four or more satellites in order to triangulate a person׳s position, and a GPS data point will typically consist of a time stamp and longitude, latitude and altitude coordinates. When worn by study participants, it enables investigators to track the mobility patterns of individuals and therefore measure environmental exposures such as time spent in the vicinity of different types of food outlet (Thornton et al., 2011). The potential applications of GPS for the study of food environments extends beyond investigating human exposure to food to identifying locations of food facilities in the environment (Fleischhacker, Evenson, Sharkey, Bell Jilcott Pitts, & Rodriguez, 2013). This is pertinent because methods used to identify food stores still have technical challenges (Hosler and Dharssi, 2010, Sharkey, 2009). Researchers have mainly relied on GIS based secondary retail food outlet databases for location information, which can introduce further misclassification if these databases provide an inaccurate representation of current food outlet locations (An and Sturm, 2012, Burgoine and Harrison, 2013, Fleischhacker et al., 2013, Liese et al., 2010).

Despite the potential of GPS to help us better understand behaviours in food environments, it is noteworthy that the existing literature detailing its application comes largely from the physical activity domain (Coombes et al., 2013, Krenn et al., 2011, Rodríguez et al., 2012) or studies that focus on travel behaviours (Axhausen et al., 2003, Chaix et al., 2012, Wolf et al., 2001), with very little from the food and diet area (Christian, 2012, Kestens et al., 2012, Zenk et al., 2011). Little is therefore known about how actual use of the environment is associated with food related behaviours (Zenk et al., 2011), and this raises the need for a better understanding of how GPS can refine current knowledge of the influence of food environments on diet and weight (Kestens et al., 2012). This is particularly the case given that has been shown that correlations between residential neighbourhoods and the places people actually visit are weak (Zenk et al., 2011).

Improving access to healthy foods is a promising strategy to prevent nutrition-related diseases; however the equivocal evidence base to date to inform such decisions poses the question of whether researchers have been measuring the food environment in the right way. This systematic review has therefore been undertaken to examine the application of GPS in studies of exposure to food environments and their potential influences on health. As far as we are aware this is the first review to specifically focus on the use of GPS in this field.

## 2 Methodology

An initial scoping exercise was undertaken in order to identify studies that: (1) were written in English and (2) were related to the use of GPS to measure factors associated with the food environment. From this initial scoping, a number of studies emerged using GPS for identifying actual location of people. The scoping exercise informed the present systematic review and suggested the studies were too heterogeneous to permit meta-analysis.

The full systematic review involved searching four electronic databases (Scopus, Medline, PubMed, and Web of Science), including reference lists of retrieved papers, and manual searches of key authors and key journals to identify relevant studies related to GPS and the neighbourhood food environment. The search keywords were: (food OR diet) AND (“global positioning systems” OR “global positioning system”). The inclusion and exclusion criteria for the systematic review were formulated as a result of the scoping exercise and a selection of papers was cross-checked by both authors. Studies were therefore included if they were written in English and if they used GPS to identify location of individuals in relation to food outlets and linked that to diet, weight status, or related health outcomes. Studies were excluded if they used GPS only to identify location of food outlets but not of people, if they were conducted in animals or used GPS for other purposes than measuring use of, or exposure to, food environments (such as agriculture and farming, physical activity and sports, alcohol behaviours, travel behaviours other than to purchase or consume food, etc.), or if they were not written in English. No restriction based on publication year, comparator or study design was applied. The final cut-off date for inclusion in the review was January 2015.

The included studies were appraised against a set of nine quality criteria: (1) representativeness of the sample population (determined based on the information stated in each paper reviewed by comparing the stated target population with the sample detailed along with any discussion of sample representativeness); (2) sample size; (3) length of GPS recording period; (4) how many food outlet types were assessed; (5) if a dietary or (6) an anthropometric measure was included; (7) if positional accuracy was reported; (8) data quality (such as whether the dietary outcome was linked to the GPS location); and (9) if the study had been subjected to peer review. These criteria were developed from those previously used in a systematic review of the use of GPS in physical activity research (Krenn et al., 2011). Additional quality criteria that were included in this review were whether studies included analysis with dietary and anthropometric factors, as we wanted to observe if measuring the food environment and diet or weight in different ways might lead to equivocal associations. The length of GPS recording period was pertinent here included as it may be that a substantial number of recording days are required to capture habitual food-related behaviours (Kerr et al., 2011). We also assigned a higher quality score to studies that looked at more food outlet types. Our rationale was that food is purchased from a range of sources and even foods that appear to be purchased from fast food outlets (i.e. burger and chips) often actually come from alternative outlet types. Hence even if the focus is on a single type of food (e.g. fast food) or outlet type (e.g. fast food outlets) then better quality studies would consider them in the context of other outlets types.

The quality of each paper was depicted by a score summarising the metrics to provide an overall impression of the quality of the available evidence. A weighting system was employed whereby the score for each metric was divided by the maximum possible value so that each metric had the same weighting in the overall quality score. The scores were initially assigned by the first author and cross-checked by the second with disagreements being resolved by discussion. The way each quality criterion (adapted from Krenn et al. (2011)) was assigned is detailed as a footnote to Table 2, with the criteria ranging from 0 or 1 (lowest quality: the sample was not representative, GPS tracking was undertaken for less than 2 days, anthropometric measures were self-reported, GPS data quality was not discussed etc.) to up to 3 (highest quality: the sample was representative, GPS tracking was undertaken for over 4 days, anthropometric measures were objectively measured, GPS data quality was discussed etc.).

**Table 1:** General description of studies

**Attribute**	***N* (count)**	**Studies**
***Year of publication***		
2011	1	Zenk et al. (2011)
2012	2	Christian, 2012, Huang et al., 2012
2013	1	Gustafson et al. (2013)
2014	2	Harrison et al., 2014, Shearer et al., 2014
		
		
***Setting***		
USA	4	Christian, 2012, Gustafson et al., 2013, Huang et al., 2012, Zenk et al., 2011
Canada	1	Shearer et al. (2014)
UK	1	Harrison et al. (2014)
		
		
***Model (type) of GPS receiver used***		
Garmin Foretrex 201 (SiRF Star II chipset)	1	Zenk et al. (2011)
Qstarz BT-1000XT	4	Christian, 2012, Gustafson et al., 2013, Harrison et al., 2014, Huang et al., 2012
EM-408 (SiRFstar III)	1	Shearer et al. (2014)
		
		
***System used to classify the types of food outlets***		
NAICS (North America Industry Classification System)	1	Gustafson et al. (2013)
SIC (Standard Industrial Classification)	1	Shearer et al. (2014)
Other: (Lake, Burgoine, Greenhalgh, Stamp & Tyrrell, 2010) typology	1	Harrison et al. (2014)
Own	3	Christian, 2012, Huang et al., 2012, Zenk et al., 2011
		
		
***Number of food outlet types assessed***		
2–4	3	Christian, 2012, Shearer et al., 2014, Zenk et al., 2011
Over 6	3	Gustafson et al., 2013, Harrison et al., 2014, Huang et al., 2012
		
		
***Types of food outlets***		
Supermarket or grocery store	6	Christian, 2012, Gustafson et al., 2013, Harrison et al., 2014, Huang et al., 2012, Shearer et al., 2014, Zenk et al., 2011
Specialty store	2	Gustafson et al., 2013, Harrison et al., 2014
Fast food outlet	6	Christian, 2012, Gustafson et al., 2013, Harrison et al., 2014, Huang et al., 2012, Shearer et al., 2014, Zenk et al., 2011
Restaurants	4	Christian, 2012, Harrison et al., 2014, Huang et al., 2012, Shearer et al., 2014
Farmers’ market	2	Gustafson et al., 2013, Huang et al., 2012
Convenience store (including gas stations)	5	Christian, 2012, Gustafson et al., 2013, Harrison et al., 2014, Huang et al., 2012, Shearer et al., 2014
Markets	1	Christian (2012)
Corner store	1	Huang et al. (2012)
Supercentre	1	Gustafson et al. (2013)
Produce stand	1	Gustafson et al. (2013)
Other food outlet types (such as discount stores, beverage stores, food bank, cafe)	2	Harrison et al., 2014, Huang et al., 2012

**Table 2:** Quality appraisal—studies of the use of/exposure to the food environment.

Study	Representativeness^a^	Sample size^b^	Length of recording^c^	Variety of food outlet types d	Dietary component^e^	Anthropometric component^f^	Positional accuracy reported^g^	GPS data quality discussed^h^	Peer reviewed^i^	Total weighted score
1. Christian (2012), US	0	2	1	2	0	0	0	0	1	3.2
2. Gustafson et al. (2013), US	0	2	1	3	0	0	0	0	1	3.5
3. Huang et al. (2012), US	1	0	1	3	n.e.	n.e.	0	0	1	3.5
4. Zenk et al. (2011), US	0	2	2	2	0	n.e.	0	1	1	4.7
5. Harrison et al. (2014), UK	0	2	2	3	n.e.	n.e.	1	1	1	6
6. Shearer et al. (2014), Canada	0	2	2	2	0	n.e.	1	1	1	5.7

Quality criteria for use of / exposure to the food environment studies.

This criteria was adapted from Krenn et al. (2011).

n.a.=Not applicable; n.e.=not examined.

a The sample was representative of the selected target group (as stated in the paper): 0=no, 1=yes.

b Sample size for the GPS study: 0=<=50, 1=51–100, 2=>100.

c Recording period: 0=<=2 days, 1=3–4 days, 2=>4 days.

d Asses a variety of food outlet types: 1=1 food outlet type; 2=2–4 food outlet types; 3=5 or more food outlet types.

e Measure of dietary outcome: 0=frequency questionnaire (consumption (FFQ) or habitual food purchase), 1=food diary, 2=objective measure (nutrient intake etc.).

f Anthropometric measures: 0=self-reported; 1=measured.

g Positional accuracy of the device used was reported: 0=no, 1=yes.

h GPS data quality discussed: 0=not discussed, 1=data quality discussed (does the paper reflect on GPS issues such as signal loss, is dietary outcome linked to GPS location?).

i The study was published in a peer-reviewed journal/book: 0=no, 1=yes.

## 3 Results

### 3.1 Study selection

**Fig. 1:**
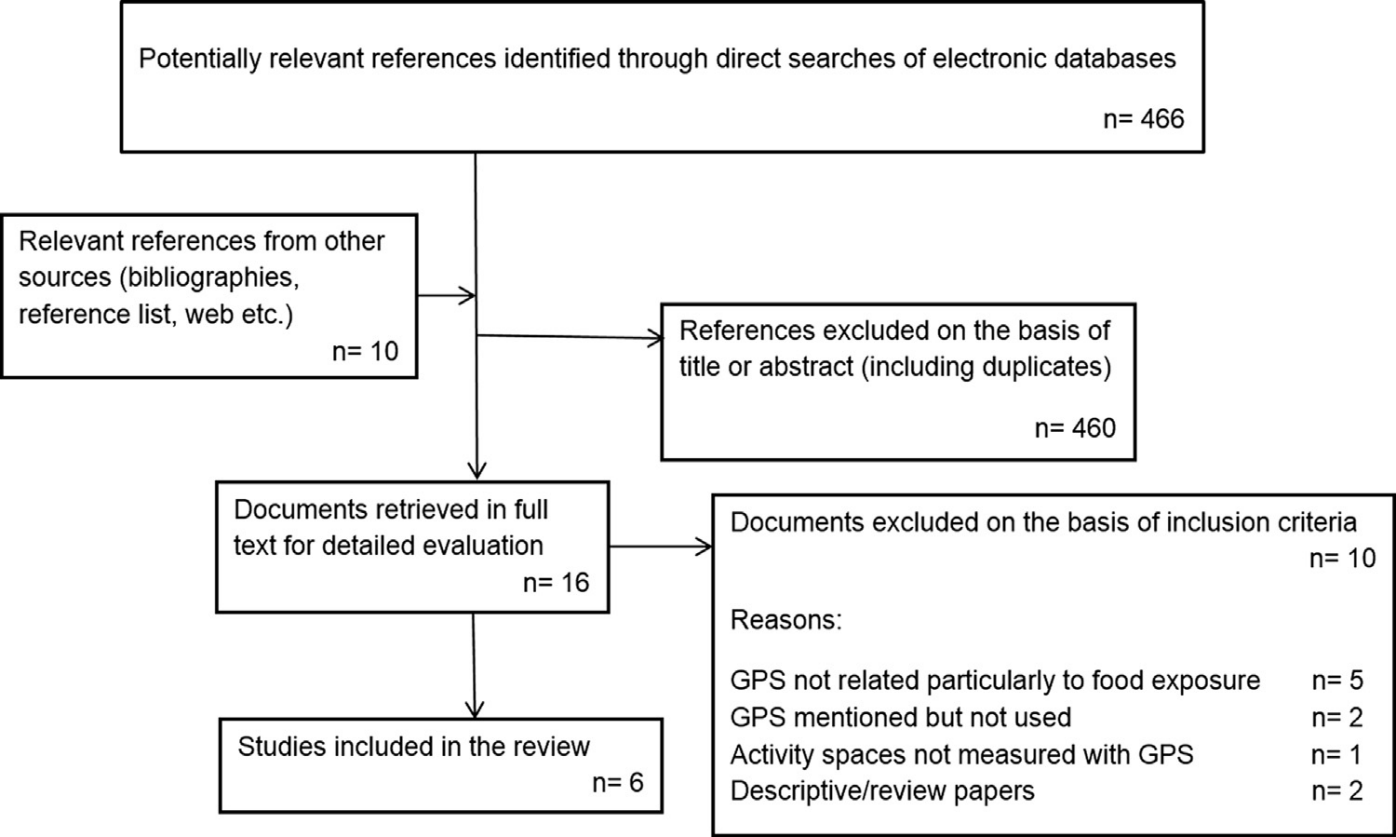
Study flowchart.

Overall, 466 potentially relevant publications were identified based on title and an additional 10 were found by checking the reference lists of the included papers (Fig. 1). Examination of abstracts resulted in the exclusion of 460 articles. The full text of 16 papers was assessed, and 10 were found not to meet the inclusion criteria. This was mostly because there was either no mention of GPS or GPS was briefly mentioned but not used in the study, no mention of food, diet, or other related health behaviours, or the studies were simply describing the literature in a conceptual way rather than mapping the environment or examining associations with health outcomes. The review process ultimately identified a small number of final relevant studies (n=6) that were published between 2011 and 2014 (Table 1, Table 2, Appendix A).

### 3.2 Quality of studies

The overall quality score for each study had the potential to range between 0 and 14. Actual scores of studies ranged from 3.2 to 6 (Table 2). While no studies were situated in the upper third of the scale, three studies were in the middle third and three studies in the lower third. Overall, the studies included in this review can be regarded as being of moderate quality.

### 3.3 Description of studies

Unsurprisingly, most studies came from the USA (*n*=4), with only one study from Canada and one from the UK. Qstarz BT-1000XT models were the most commonly used GPS receivers (*n*=4). While three studies developed their own system of classifying food outlets, the other three used a pre-established validated classification system. In terms of the variety of food outlet types assessed, studies looked at between 2 and 13 food outlet types, with the most common ones being supermarket or grocery store, fast food outlet, restaurant and convenience store (Table 1).

Sample sizes ranged from 35 to 380 participants. Most studies were focused on adults (*n*=4), with one of the studies looking only at people aged over 45 and one at people over 50 with mobility disabilities; only in 2014 have studies started to emerge looking at children or adolescents (*n*=2). Four studies reported participation or enrolment rates: 11% (Gustafson et al., 2013), 28% (Zenk et al., 2011), 27% (Shearer et al., 2014) a 27%, and 77% (Harrison, Burgoine, Corder, van Sluijs, & Jones, 2014). For the adult studies, recruitment was undertaken through flyers (*n*=3) (Christian, 2012, Gustafson et al., 2013, Huang et al., 2012), neighbourhood association meetings (Christian, 2012), announcements in relevant organisational e-newsletters (Huang et al., 2012), telephone (Zenk et al., 2011); for adolescent studies, recruitment was undertaken through presentation in schools and distribution of packages in which parental and student consent were included (Harrison et al., 2014, Shearer et al., 2014).

The GPS recording period was 3 days for half of the studies (Christian, 2012, Gustafson et al., 2013, Huang et al., 2012) and 7 days for the other half (Harrison et al., 2014, Shearer et al., 2014, Zenk et al., 2011). In 5 studies (Gustafson et al., 2013, Harrison et al., 2014, Huang et al., 2012, Shearer et al., 2014, Zenk et al., 2011), GPS measurement was made on both weekdays and weekend days, whereas one study (Christian, 2012) trimmed the GPS data to the first three weekdays only. The reasons given for limiting the activity space data to three days were that it eliminates the need for participants to charge the GPS and it facilitates measurement of a set of local retail food opportunities, rather than actual food shopping behaviours.

Three of the studies reported the number of participants that remained from the initial sample size to the analysis stage: between 2% and 17% of participants were lost in the process. The various reasons why data was excluded from the analysis were: trips without eligible GPS data, participants did not wear the GPS for the entire required length of time (Christian, 2012, Gustafson et al., 2013) or at all (Zenk et al., 2011), there were unknown routes between destinations due to reception issues (Christian, 2012), the participants travelled outside the study area (Christian, 2012, Zenk et al., 2011), there were data collection errors by staff (Zenk et al., 2011), or data was “suspicious”, a term not clarified in the paper but confirmed by the authors to represent sparse data (Zenk et al., 2011). While studies commented on issues such as battery life (Christian, 2012, Shearer et al., 2014, Zenk et al., 2011) (*n*=3), time to first GPS location fix (Christian, 2012, Shearer et al., 2014) (*n*=2), and interval of time at which GPS records location (Christian, 2012, Harrison et al., 2014, Shearer et al., 2014, Zenk et al., 2011) (*n*=4) ranging between 1 and 30 s, only two most recent studies (Harrison et al., 2014, Shearer et al., 2014) commented on positional accuracy of the GPS device, one (Shearer et al., 2014) reporting the sensitivity of the GPS receiver, and one (Harrison et al., 2014) reporting signal drift resulting in the loss of less than 1% of GPS points. Four studies (Christian, 2012, Harrison et al., 2014, Shearer et al., 2014, Zenk et al., 2011) gave additional detail on the GPS data, such as how the participants were instructed to wear or charge the device, how many points the device yielded, how these were treated and analysed.

#### 3.3.1 Food and weight related outcomes

Retrospective questionnaires and immediate diary records of an individual׳s dietary behaviours are attractive because they offer simple and inexpensive estimates of habitual behaviours. Most studies looking at dietary behaviours to date rely on such reports (Diez-Roux et al., 1999, Yang et al., 2010) and the majority of the studies included in this review (*n*=4) used food consumption or food purchase frequency questionnaires (Christian, 2012, Gustafson et al., 2013, Zenk et al., 2011, Shearer et al., 2014), while one study (Huang et al., 2012) used semi-structured interviews. The Harvard Youth/Adolescent Questionnaire used in Shearer et al. Shearer et al. (2014) was the only questionnaire that was reported to be validated. Four of the studies (Christian, 2012, Gustafson et al., 2013, Shearer et al., 2014, Zenk et al., 2011) assessed self-reported dietary outcomes: all four investigated frequency of consumption of specific foods, while Zenk et al. (2011) also looked at mean daily saturated fat intake in grams and servings of specific foods, and Shearer et al. (2014) also examined caloric intake and diet quality. Two of these (Christian, 2012, Gustafson et al., 2013) also assessed frequency of purchase, and one (Gustafson et al., 2013) studied food venue choice. Harrison et al. (2014) did not investigate associations with diet.

While four studies (Christian, 2012, Gustafson et al., 2013, Shearer et al., 2014, Zenk et al., 2011) were focused on how measures of food accessibility or availability relate to weight or dietary behaviours, one study (Huang et al., 2012) focused on how older people with mobility disabilities access locations, travel mode, and what the facilitators and barriers to accessing locations outside the home may be. Four studies did not examine any anthropometric measures (Harrison et al., 2014, Huang et al., 2012, Shearer et al., 2014, Zenk et al., 2011), whilst two included self-reported BMI (Christian, 2012, Gustafson et al., 2013). Christian et al. Christian (2012) reported weight status as a categorical outcome (underweight/normal for BMI <25, overweight for 25<=BMI<30, and obese for BMI>=30). Gustafson et al. (2013) also reported BMI as categorical (underweight, normal weight, overweight, obese), but it is used to describe the sample rather than as an outcome. None of the studies used objectively measured weight.

#### 3.3.2 Environmental exposure assessment

All studies were concerned with exposure to food venues in the activity space. The activity space was measured in different ways. Zenk et al. (2011) adapted two measures from the existing literature, calculating a one standard deviation ellipse and a daily path area. The daily path area was calculated by buffering all GPS points by 0.5 mile (800 m) and merging (dissolving) these separate features into one space. Two papers published after (Zenk et al., 2011) use the same distance when calculating activity space based on daily path area (Christian, 2012, Gustafson et al., 2013); the reason for using this distance was that Zenk et al. (2011) noted significant associations using it, and preliminary analysis in one of the studies (Christian, 2012) found no associations when using a 0.25 mile buffer. Shearer et al. (2014) used a more restricted distance (50 m) for the daily path area, based on the logic that the distance would include any food outlet located on either side of a road as part of a participant׳s activity space. Harrison et al. (2014) used a slightly larger 100 m buffer around GPS routes from home to school and counted the number of food outlet facilities within these buffers in order to evaluate exposure to food on each route. One study (Huang et al., 2012) did not use a direct measure of activity space; GPS locations were used as a discussion starting point for where study participants went while wearing the GPS. The authors reported that GPS provided additional objective information on what types of facilities and venues people access most.

Most studies (*n*=5) (Christian, 2012, Gustafson et al., 2013, Harrison et al., 2014, Shearer et al., 2014, Zenk et al., 2011) utilised the ArcGIS software package for calculating spatial access to and availability of environmental characteristics. Environmental attributes measured ranged from counts, proportions and density of food outlets within the daily activity space (Christian, 2012, Gustafson et al., 2013, Harrison et al., 2014, Shearer et al., 2014, Zenk et al., 2011) to audits of food stores (Gustafson et al., 2013). Some looked beyond food environments at environmental attributes related to physical activity, such as neighbourhood walkability (Huang et al., 2012), parkland use (Zenk et al., 2011) or access to physical activity facilities on the route to school (Harrison et al., 2014). While all studies focused on the activity space environment, four (Christian, 2012, Harrison et al., 2014, Shearer et al., 2014, Zenk et al., 2011) also compared the activity with the neighbourhood based food environment. In Zenk et al. (2011) the neighbourhood food environment was defined as the number of food outlets of each type in each residential neighbourhood (0.5 mile street-network buffer around the census block centroid). Christian (2012) calculated a neighbourhood-level measure defined as either density (food outlets of each type per square mile or per ten square miles) or proportion (percentage of food outlets among all food stores). Shearer et al., (2014) employed a ‘home-based’ approach by looking at counts and average distance to food outlet locations from the home origin within 1 km network-based buffers. Harrison et al., (2014) counted the number of food outlets within 100 m network-based routes from home to school.

### 3.4 Main findings of the studies included

Associations between activity space as well as neighbourhood food environment and diet related outcomes were equivocal across the small sample of studies included in this review. Three studies found associations between activity based food environment measures based on the daily path area and some dietary components (Christian, 2012, Shearer et al., 2014, Zenk et al., 2011), but not others (Gustafson et al., 2013, Shearer et al., 2014, Zenk et al., 2011); there was an inverse association reported between the identification of unhealthy food dense activity spaces and whole grain intake (Christian, 2012, Zenk et al., 2011) and fruit and vegetable consumption (Shearer et al., 2014), with a positive association with saturated fat intake (Zenk et al., 2011), but no significant associations were found with fruit and vegetable intake (Zenk et al., 2011), added sugar, red meat or fried potatoes (Christian, 2012). Activity space measures were also associated with the availability of specific foods in a food venue (Gustafson et al., 2013), which suggests it is not merely the presence of food outlets that influence behaviour, but the availability of food types within that outlet. Additionally, greater accessibility of calorically dense, ready-to-eat foods in the activity space was associated with higher weight status (Christian, 2012). Regarding associations with neighbourhood-based environments, Zenk et al. (2011) found no associations between residential neighbourhood based fast food exposures and dietary intakes. Similarly, Shearer et al. (2014) found no association between home-based food exposure measures and diet.

The studies that examined the differences between GPS measured activity-space and neighbourhood-based food environment exposures reported stark differences between the two measurement approaches. Shearer et al. (2014) found that home-based measures overestimate the importance of the residential neighbourhood, with a smaller number of food locations available for neighbourhood than for GPS-based measures, especially in rural areas. Christian (2012) and Zenk et al. (2011) reported weak associations between neighbourhood- and activity space-based food environment measures. This highlights how the residential neighbourhood is likely to be a poor proxy for the food environment to which individuals are exposed through the course of their day-to-day activities. Indeed, one study (Christian, 2012) showed that individuals encountered very different food environments in their daily travel than that within or near their neighbourhood. Furthermore, Harrison et al. (2014) also found that GIS modelled routes do not capture actual environmental exposures particularly well, especially for pedestrians.

### 3.5 Data quality

Although the small sample size (6 studies) precluded formal statistical testing, it was observed that sample size, participant age, GPS manufacturer and number of food outlet types analysed were not associated with data quality. Furthermore, for the three studies for which this information was available (Christian, 2012, Gustafson et al., 2013, Zenk et al., 2011), there was no relationship between the number of days for which participants were asked to wear the GPS and data loss (measured in number of participants lost from the initial sample). There was some evidence suggestive of an association between data quality and year of publication, with quality scores ranging from 6 (Harrison et al., 2014) and 5.7 (Shearer et al., 2014) for studies published in 2014, to 3.2 (Christian, 2012), 3.5 (Gustafson et al., 2013, Huang et al., 2012) and 4.7 (Zenk et al., 2011) for earlier studies.

## 4 Discussion

### 4.1 Problems and considerations in the use of GPS in food environment studies

Characterising exposure to the retail food environment as accurately as possible is important for many reasons, including identifying areas with limited retail access and therefore pushing policy strategies to reduce inequalities and nutrition-related diseases by improving access to healthy food. To this end, GPS technologies may be increasingly useful. However, their use should be carefully weighed against their limitations depending on the study scale and context.

It was observed that most studies included in this review come from the US, and only one from the UK. This is pertinent given the fact that in the US the contrast in urban design and neighbourhood segregation may lead to a different importance of the food environment compared to the UK. Furthermore, most studies have been conducted in adult populations, whilst it is known that children relate to their environment differently (Brembeck et al., 2013), so the food environment may therefore have a different importance in this population.

Physical activity studies typically temporally link information on activity levels recorded with accelerometer devices with the locations people visit throughout the day. There is the potential to improve specificity of measurement using similar methods in food environment studies if temporally-linked food diaries or ecological momentary assessment techniques can be used to derive time-dependent measures of eating occasions or food purchases. However, this has not been attempted thus far. The most common method used in the literature has been the food frequency questionnaire (FFQ), yet it has been shown that food diaries have revealed relationships not observed in the FFQ (Freedman et al., 2006), and that FFQs show only weak associations with dietary biomarkers (Kristal, Peters & Potter, 2005). That is at least in part because food diaries can be completed at the same time as GPS data is collected and are able to record times when certain food items have been eaten. They can therefore be temporally linked to the GPS exposures, while FFQs assess habitual food intake over a longer period of time, are subject to recall bias, and are not suitable for linking with temporal movement patterns. These observations may in part explain the equivocal associations with diet-related outcomes found in the studies.

While GPS are becoming the gold standard for determining continuous location with high geospatial accuracy, Liese et al. (2010) call for caution as they are subject to error that can arise from satellite-related errors, signal propagation errors and receiver errors. It is noteworthy that physical activity studies appear more likely to discuss issues such as location precision, data loss and GPS data quality (Krenn et al., 2011); in this review three studies touch upon GPS signal loss and reasons why, and only two most recent studies discuss positional accuracy of the GPS device used. As technology progresses there is the increasing potential to attain positional augmentation using coordinates collected from a mobile phone or radio frequency identification tags that can provide at least partial solutions for technical issues such as signal loss. Further, computational algorithms that have started to emerge to eliminate spurious GPS points and identify travel mode of interest. The collection of GPS data also requires technical knowledge, and challenges such as signal loss, delay in acquiring satellite signal after start-up, precision of the device, battery power, or participants forgetting to switch on the device remain (Thornton et al., 2011). For these reasons, cleaning protocols have been developed to attempt to overcome these issues (Oliver et al., 2010, Schüssler and Axhausen, 2008). Although in this very small sample of studies GPS model was not linked to data quality, modern devices are becoming quicker to pick up signal and suffer less data loss than older models. A recent study (Duncan et al., 2013) comparing seven models of GPS devices reported that the Qstarz BT-1000XT had the longest battery life, the lowest circular error probability (a metric commonly used to quantify GPS accuracy) and the lowest acquisition time (time to first fix). It is has recently been reported that Qstarz devices are generally acceptable for use in large population health studies, especially with relatively long data collection periods of over 7 days (Schipperijn et al., 2014).

Caution must be taken in inferring causality when studying human behaviour with the help of GPS, as it cannot be determined if food related activity patterns in the neighbourhood are a cause or consequence of the food environment (Zenk et al., 2011). This is because individuals who want to consume a particular type of food may seek out environments with higher concentration of that food type in order to obtain it, which might preclude causal inference. This has been termed ‘daily selective mobility bias’ (Chaix et al., 2013). Despite this, characterizing the space within which people move or travel during the course of their day-to-day activities rather than only where they live, work or study, clearly offers the potential to provide a more comprehensive and accurate assessment of the environment to which individuals are exposed and utilise (Zenk et al., 2011) and facilitates the detection of temporal and spatial patterns of behaviours that relate more closely to health outcomes of interest (Kerr et al., 2011).

### 4.2 Strengths and limitations

To our knowledge this is the first systematic review to identify studies that investigate exposure to the food environment with the help of GPS. The strengths of this review include the systematic methods used for assessing the quality of studies by more than one reviewer. It provided an overall summary of the quality of evidence available and reported important technical aspects of the GPS assessment in detail. The quality criteria developed, although based on a previously-published one by Krenn et al. (2011), are however not without limitations. A limitation of this review is the fact that only papers written in English were considered and relevant material written in foreign languages may be omitted. Furthermore, conclusions need to be interpreted in the context of the small number of studies retrieved, which also contributed to the fact that no meta-analysis was possible in this instance, as the small sample of heterogeneous studies provided little power to detect associations.

## 5 Conclusion

This review has shown that the use of GPS to measure exposure to the food environment is still in its infancy and there are considerable variations in the methods and techniques used, with several issues related to data collection, accuracy, behaviour classification and analysis (Boruff et al., 2012) that need to be carefully considered. Very few food environment studies to date have used GPS, and this is especially so in children and outside the US. There are clearly also a number of outstanding methodological and practical issues associated with their application. However, GPS technology is improving, and the findings from the few studies that have attempted to use the technology illustrate the potential added value that can be obtained from being able to record and analyse patterns of mobility in the food environment.

## Contributors

All authors had full access to the data and can take responsibility for the integrity of the data and the accuracy of the data analyses. AC performed data extraction and synthesis and wrote all drafts of the paper. AJ advised at all stages, cross-checked data quality and contributed to all drafts of the paper and approved the final version.

## Funding

AC was funded by the lord Zuckerman Ph.D. scholarship. APJ was partially supported by the Centre for Diet and Activity Research, a UK Clinical Research Collaboration Public Health Research Centre of Excellence. Funding from the British Heart Foundation, Department of Health, Economic and Social Research Council, Medical Research Council, and the Wellcome Trust, under the auspices of the UK Clinical Research Collaboration, is gratefully acknowledged.

The funding sources had no role in the design and conduct of the study or in the collection, management, analysis, and interpretation of the data.

## Conflict of interest

All authors have completed the conflict of interest form at http://elsevier6.custhelp.com/ci/fattach/get/3199579/0/filename/Example_Conflict+of+Interest+Form.pdf (available on request from the corresponding author) and declare that (1) Neither have support from any company for the submitted work; (2) neither have any relationships with companies that might have an interest in the submitted work in the previous 3 years; (3) their spouses, partners, or children have no financial relationships that may be relevant to the submitted work; and (4) Both have no non-financial interests that may be relevant to the submitted work.

## Data sharing

Data sharing: no additional data available.

## Footnotes

Appendix A Supplementary data associated with this article can be found in the online version at doi:10.1016/j.ssmph.2016.04.001.
