# Supplementary material for: How can GPS technology help us better understand exposure to the food environment? A systematic review
Source: SSM Popul Health. 2016 Apr 18;2:196–205. doi: 10.1016/j.ssmph.2016.04.001 (PMC5165043; doi:10.1016/j.ssmph.2016.04.001)
Supplement: Supplementary file 1 — Supplementary material [file mmc1.doc]

**Appendix A. Description of included studies**

| **Study** | **Subjects** | **Objectives** | **Dietary measure (food intake/**  **purchase)** | **Anthropo-**  **metric measure** | **Other covariates** | **GPS device** | **Time period of GPS wear** | **Activity space calculation** | **GPS Data loss (DL) + problems (P) + solutions (S)** | **Secondary data source used** | **Food classification scheme used** | **Food locations (food outlets, food stores, food providers)** | **Environmental analysis/attributes** |
| --- | --- | --- | --- | --- | --- | --- | --- | --- | --- | --- | --- | --- | --- |
| ([Christian, 2012](#_ENREF_1)), US | N:121 (representing about 4.9% of eligible adults  in the target area); N:101 participants with complete three-day GPS tracks  Age: 18-65  Gender: 56.4% female (out of 101) | (1) How do individuals’ activity-based  measures of food accessibility compare to neighbourhood based  measures?  (2) How do these activity-based measures  relate to individual characteristics, including weight?  (3) Are activity-based measures associated with diet and food purchasing? | Survey on diet (FFQ) and food purchase (frequency) | BMI, self-reported, categorical (underweight/normal for BMI < 25, overweight for  BMI>=25, obese for BMI>=30) | Age, gender, race, household income, education, employment, marital status | Qstarz BT-1000XT Travel Recorder | 3 weekdays | ArcGIS 10: Euclidean buffer of 0.50 miles (2640 feet) around  participant’s three-day GPS track line. | DL: approx. 17% lost from participants with not enough GPS data  P: unknown routes between destinations,  presumably due to reception issues  S: data were deemed inadequate and excluded from the  analysis | Lexington-Fayette County Health Department | Own | Food stores: 4 (supermarkets; convenience stores; FV markets; limited service restaurants (include fast food)) | -counts within daily activity buffer;  - proportion of healthy to unhealthy (RFEI scor1e) |
| ([Gustafson et al., 2013](#_ENREF_2)), US | N:121  Age: 18+  Gender:  (153 responded, 121 not eligible) | Determine the association between six various dietary indicators and 1) food venue availability;  2) food venue choice and frequency; and 3) availability of healthy food within food venue | Survey on food shopping behaviours and dietary outcomes | BMI, self reported | Age, gender, race, marital status, education, household income, employment, automobile ownership | Qstarz BT-1000XT Travel Recorder | 3 days (2 weekdays, 1 weekend) | Euclidean buffer of 0.50 miles (2640 feet) around  participant’s three-day GPS track line. | 2 people out of the 121 not eligible did not agree to wear GPS for 3 days | InfoUSA | NAICS, Own | Food stores: around 8 (**healthy**: produce stands, farmer’s markets, supermarket/grocery store; **less healthy**: supercenters, convenience stores, gas stations, fast food restaurants; specialty stores) | -counts of each venue type within activity space buffer; proportion of healthy to unhealthy (RFEI score);  -audits using Nutrition Environment Measurement Survey-Store Rudd (NEMS-S), n=22) |
| ([Huang et al., 2012](#_ENREF_4)), US | N: 35  Age: 50+ (with mobility disability)  Gender: 74.3% female | Examine: 1) where participants accessed food outside the home, 2) how they travelled to these food destinations, and 3) facilitators and barriers to food access using qualitative  interviews | n.e. (information on type of food outlets accessed) | n.e. | Age, sex, race/ethnicity, household income, driving status, neighbourhood walkability score, type of assisted device, reside in low income, reside in food desert, living in facilities that provide meals | Qstarz BT-Q1000XT | 3 days (2 weekdays, 1 weekend) | n.r. | n.r. | None, self reported in interview | Own | Food locations accessed: 13 (grocery store, full-service restaurant, other, coffee shop, fast food restaurant, food bank, senior centre, warehouse store, farmers market, convenience store, corner store, drug store, shopping mall) | -self reported barriers and facilitators and types of foods accessed;  -self-reported used transportation mode;  -neighbourhood walkability;  -food desert locator;  -self-reported PA locations used |
| ([Zenk et al., 2011](#_ENREF_7)), US | N: 131  N: 120 who wore GPS  Age: 3 categories (<45, 45-65, >64)  Gender: 75% female | Examine associations between individual and area characteristics of the environment and activity spaces, and weight related behaviour (diet, PA) | FFQ (7 days recall) (n=116-complete data) | n.e. | Age, gender, race/ethnicity, and four indicators of socioeconomic position (SEP): education, labour force participation, annual household income, and auto ownership. | Foretrex 201 (Garmin, Olathe, KS) | 7 days | ArcGIS 9.2: 2 measures: 1) standard deviation ellipse; 2) daily path area (buffering all GPS points at 0.5 mile and dissolving these separate features into a single space) | D.L.: 11 people out of 131  P: GPS data collection errors by staff (n=3); did not wear GPS or suspicious data (n=8)  S: excluded | Fast Food: county departments of agriculture, city of Detroit; supermarkets: Michigan department of agriculture | Own, based on previous literature | Food outlets: 2 (fast food outlets, supermarket) | -residential neighbourhood (0.5 mile street-  network buffer around the census block centroid);  -park land use, fast food outlets density and supermarket availability in residential neighbourhood and activity space |
| ([Harrison et al., 2014](#_ENREF_3)), | N: 175  Age: 13-14 | Compare modelled GIS and actual GPS-based routes to school in order to see how representative the modelled routes are of those actually taken | n.e. | n.e. | straight-line distance between home and school | Qstarz BT-Q1000XT waist-mounted | 7 days | GPS routes home-school:visual inspection | D.L.: <1% of recorded points | 12 district and city councils (local administrative  authorities) in Norfolk, Suffolk and Lincolnshire  in January 2012 | Six-point scheme derived from the 21-point scheme developed by ([Lake et al., 2010](#_ENREF_5)) | Food outlets: 6 (Unhealthy (takeaways, convenience stores); restaurants, supermarkets,  specialist stores, and cafes) | Counts of food outlets and physical activity facilities within 100 meter buffers around GIS and GPS routes |
| ([Shearer et al., 2014](#_ENREF_6)), Canada | N: 380  Age: 12-16 | Measure food availability and accessibility in adolescents within and outside home neighbourhoods and explore their associations with dietary intake | Dietary intake of healthy and unhealthy foods (FFQ): Harvard Youth/  Adolescent Questionnaire (YAQ) | n.e. | residential socioeconomic  status (SES) and neighbourhood type (rural, urban, suburban) | 20 channel EM-408 SiRFstar III chipset GPS receiver | 7 days | 50 m  (dissolved) buffer was generated from each participant's GPS data  resulting in a single activity space polygon |  | DMTI Enhanced  Points of Interest (EPOI) database | standard  industry classification codes (SIC) | fast food (major  fast food chains with a focus on take-away meals), restaurants (all  other types), grocery and convenience stores. | distance to and number (count) of food locations were calculated  using two approaches:  (1) home-based (network-based buffers were created using a distance of 1 km along the road network equivalent to a 15-minwalk time from the home origin)  (2) GPS-based activity space |

**n.r.** = not reported; **n.e.**= not examined; **n.a.**= not applicable; PA= physical activity; FFQ= food frequency questionnaire

**Food classification scheme used:** North America Industry Classification System (NAICS); Nutrition Environment Measures Survey (NEMS); Standard Industrial Classification (SIC); European Business Codes/ Nomenclature des Activites Economiques (NACE); Irvine-Minnesota Inventory (IMI); Lexington-Fayette County Health Department; own (developed its own definition for classifying food outlets, based on existing literature, hypothesis, chain-name recognition etc.

**Secondary databases:**

**commercial sources:** Info USA; DMTI Enhanced Points of Interest (EPOI) database of businesses and recreational points of interest for all provinces of Canada (DMTI Spatial Inc, Markham, Ont).

**government sources:** County Health Departments (US); State Department of Agriculture (US); the New York State Department of Agriculture and Markets; local administrative authorities (UK)

**References for Appendix A:**

Christian, W.J., 2012. Using geospatial technologies to explore activity-based retail food environments. Spatial and Spatio-temporal Epidemiology 3, 287-295.

Gustafson, A., Christian, J.W., Lewis, S., Moore, K., Jilcott, S., 2013. Food venue choice, consumer food environment, but not food venue availability within daily travel patterns are associated with dietary intake among adults, Lexington Kentucky 2011. Nutrition Journal 12.

Harrison, F., Burgoine, T., Corder, K., van Sluijs, E., Jones, A., 2014. How well do modelled routes to school record the environments children are exposed to?: a cross-sectional comparison of GIS-modelled and GPS-measured routes to school. International Journal of Health Geographics 13, 5.

Huang, D.L., Rosenberg, D.E., Simonovich, S.D., Belza, B., 2012. Food access patterns and barriers among midlife and older adults with mobility disabilities. Journal of Aging Research 2012.

Lake, A.A., Burgoine, T., Greenhalgh, F., Stamp, E., Tyrrell, R., 2010. The foodscape: Classification and field validation of secondary data sources. Health & Place 16, 666-673.

Shearer, C., Rainham, D., Blanchard, C., Dummer, T., Lyons, R., Kirk, S., 2014. Measuring food availability and accessibility among adolescents: Moving beyond the neighbourhood boundary. Social Science and Medicine.

Zenk, S.N., Schulz, A.J., Matthews, S.A., Odoms-Young, A., Wilbur, J., Wegrzyn, L., Gibbs, K., Braunschweig, C., Stokes, C., 2011. Activity space environment and dietary and physical activity behaviors: A pilot study. Health and Place 17, 1150-1161.
